# Supplementary material for: Impact of Diagnosis-Intervention Packet (DIP) reforms on inpatient services for low-income populations in central China: A multi-stage interrupted time-series analysis
Source: PLoS One. 2025 May 22;20(5):e0323194. doi: 10.1371/journal.pone.0323194 (PMC12143900; doi:10.1371/journal.pone.0323194)
Supplement: S1 Table — (DOCX) [file pone.0323194.s001.docx]

**Table S1. Robustness tests.**

| hospitals levels | **β_1_** | **β_2_** | **β_3_** | **β_4_** | **β_5_** | **β_0_** |
| --- | --- | --- | --- | --- | --- | --- |
|  | Estimate(95%CI) | Estimate(95%CI) | Estimate(95%CI) | Estimate(95%CI) | Estimate(95%CI) | Estimate(95%CI) |
| **Total hospitalization costs** | | | | | | |
| Total hospitals | -0.004(-0.012,0.004) | 0.111(0.004,0.217)* | 0.005(-0.011,0.022) | -0.035(-0.141,0.072) | -0.003(-0.017,0.011) | 8.028(7.946,8.110)*** |
| Tertiary hospitals | -0.029(-0.041,-0.017) | 0.098(-0.071,0.267) | 0.032(0.071,0.267)* | 0.076(-0.091,0.243) | -0.002(-0.024,0.020) | 9.334(9.212,9.455)*** |
| Secondary hospitals | 0.001(-0.007,0.009) | 0.052(--0.045,0.149) | -0.003(-0.020,0.014) | -0.015(-0.113,0.084) | 0.001(-0.013,0.015) | 8.470(8.385,8.555)*** |
| Primary hospitals | 0.007(-0.001,0.014) | 0.048(-0.040,0.135) | -0.012(-0.028，0.004) | -0.024(-0.113,0.065) | 0.011(-0.002,0.024) | 7.307(7.226,7.388)*** |
| **Out-of-pocket ratios** | | | | | | |
| Total hospitals | 0.370(0.216,0.525)*** | -0.912(-2.759,0.936) | -0.349(-0.709,0.011) | 0.116(-1.791,2.023) | -0.212(-0.543,0.119) | 6.590(4.628,8.552)*** |
| Tertiary hospitals | 0.544(0.239,0.850)** | -0.599(-3.496,2.297) | -0.442(-1.070,0.186) | 0.033(-2.918,2.984) | -0.237(-0.763,0.289) | 10.883(7.507,14.260)*** |
| Secondary hospitals | 0.436(0.245,0.628)*** | -1.417(-3.586,0.753) | -0.426(-0.868,0.017) | 0.227(-2.013,2.466) | -0.193(-0.600,0.213) | 8.769(6.328,11.210)*** |
| Primary hospitals | 0.288(0.161,0.415)*** | -0.648(-0.215,0.854) | -0.287(-0.581,0.007) | 0.145(-1.406,1.696) | -0.140(-0.410,0.130) | 3.849(2.244,5.455)*** |
| **30-Day readmission rates** | | | | | | |
| Total hospitals | -0.289(-0.414,-0.163)*** | 1.042(-1.429,3.513) | 0.295(-0.151,0.0741) | 0.147(-2.477,2.771) | 0.174(-0.256,0.605) | 17.946(16.464,19.429)*** |
| Tertiary hospitals | -0.430(-0.668,-0.192)** | 1.223(-3.491,5.937) | 0.550(-0.299,1.398) | 0.802(-4.203,5.808) | 0.076(-0.743,0.896) | 26.519(23.714,29.324)*** |
| Secondary hospitals | -0.378(-0.545,-0.211)*** | 1.282(-1.373,3.936) | 0.305(-0.046,0.655) | 0.437(-2.149,3.023) | 0.329(0.014,0.645)* | 20.406(18.736,22.076)*** |
| Primary hospitals | -0.208(-0.311,-0.106)*** | 0.986(-0.982,2.953) | 0.186(-0.072,0.444) | -0.202(-2.210,1.807) | 0.233(-0.013,0.479) | 13.842(12.635,15.050) |
| **Length of stay** | | | | | | |
| Total hospitals | -0.103(-0.160,-0.047)** | 0.333(-0.524,1.190) | 0.055(-0.063,0.174) | 0.168(-0.674,1.010) | 0.034(-0.071,0.139) | 10.780(10.210,11.350)*** |
| Tertiary hospitals | -0.151(-0.217,-0.084)*** | -0.061(-1.137,1.016) | 0.069(-0.070,0.208) | 0.630(-0.413,1.674) | 0.058(-0.069,0.184) | 13.179(12.517,13.840)*** |
| Secondary hospitals | -0.176(-0.265,-0.087)*** | 0.426(-0.920,1.772) | 0.098(-0.088,0.284) | 0.168(-1.155,1.491) | 0.075(-0.091,0.240) | 13.180(12.281,14.079)*** |
| Primary hospitals | -0.025(-0.068,0.018) | 0.173(-0.460,0.807) | -0.006(-0.096,0.084) | 0.151(-0.474,0.777) | 0.027(-0.052,0.106) | 8.361(7.923,8.798)*** |

Note: * *p*<0.05; ***p*<0.01; *** *p*<0.001.
