# Supplementary material for: Impact of Diagnosis-Intervention Packet (DIP) reforms on inpatient services for low-income populations in central China: A multi-stage interrupted time-series analysis
Source: PLoS One. 2025 May 22;20(5):e0323194. doi: 10.1371/journal.pone.0323194 (PMC12143900; doi:10.1371/journal.pone.0323194)
Supplement: Attachment — Submitted filename: Response to Reviewers.docx [file pone.0323194.s003.docx]

**Response to editor：**

**1. Please ensure that your manuscript meets PLOS ONE's style requirements, including those for file naming.**

- Thank you for your valuable feedback. I have carefully reviewed and confirmed that the manuscript now fully adheres to PLOS ONE's style requirements. Specifically, I have ensured that the line spacing, heading levels, font type, and size align with the guidelines provided. Additionally, I have renamed the uploaded file as per your instructions.

If there are any further formatting concerns, please feel free to let me know.

Thank you once again for your assistance.

**2. We note that you have indicated that there are restrictions to data sharing for this study. For studies involving human research participant data or other sensitive data, we encourage authors to share de-identified or anonymized data. However, when data cannot be publicly shared for ethical reasons, we allow authors to make their data sets available upon request.**

- Thank you for your inquiry regarding data availability for my manuscript. I understand the importance of data sharing, and I would like to provide clarification on the restrictions associated with the dataset used in this study.

a) Ethical and Legal Restrictions on Sharing Data:

The dataset used in this study is provided by the Hubei Provincial Medical Security Bureau, and the data include sensitive patient information. As such, I do not have the rights to share this de-identified data publicly. The data are subject to ethical and legal restrictions imposed by the local ethics committee and the relevant regulatory bodies. Specifically, the data contain potentially identifying patient information, and sharing it would violate the privacy agreements under which the data were collected.

b) Data Access Committee:

While I cannot share the data directly, researchers who are interested in accessing the data may contact the Hubei Provincial Medical Security Bureau for further information on how to apply for data access. I would be happy to provide any further details or assist in directing interested parties to the appropriate contacts.

c) Data Availability Statement Update:

Since I am unable to share the dataset directly, I have updated the Data Availability Statement in the submission form to include the necessary contact information for data access requests, as outlined above.

**Data Availability Statement:**

The data that support the findings of this study were obtained from the Hubei Provincial Medical Security Bureau. Due to legal restrictions under China’s Personal Information Protection Act (2021) and the Regulations on the Supervision and Administration of Medical Insurance Fund Use (2021), which mandate strict protection of patient privacy and healthcare data security, the raw dataset cannot be made publicly available. Researchers interested in accessing the de-identified data can apply by submitting a formal request to the Hubei Provincial Medical Security Bureau (contact email: [ybjysq@hubei.gov.cn]). Applications must include a detailed research proposal, ethical approval documentation from the applicant’s institution, and a signed data use agreement prohibiting redistribution or commercial use. Approved researchers can access the data only within the secure facilities of the platform; external data transfer is prohibited. The authors confirm that they had no special privileges in accessing the data, and all analyses were conducted in compliance with the platform’s governance protocols.

I hope this explanation addresses the journal’s concerns regarding data availability. Should you need any additional information or clarification, please feel free to let me know.

**3.Additional Editor Comments: The reviewers pointed out several issues in their comments. I agree with them. Please rewrite the text and justify any suggestions you don't accept.**

We would like to express our sincere gratitude to you and the reviewers for your thorough review and valuable suggestions! We have made significant revisions to the paper based on the reviewers' comments, with particular attention to rewriting and enhancing the content of core chapters such as the abstract, introduction, and discussion. We have addressed each of the reviewers' concerns in detail. Below is a summary of the revisions:

**1. Main Revisions**

1) **Abstract**: In response to the reviewers' requests, we expanded the background section to include a discussion on the challenges posed by escalating global healthcare costs and health equity. We also clarified the unique role of China's DIP reform in controlling costs and promoting equity. The research objectives were then refocused, emphasizing the study's goal of "assessing the heterogeneous impact of DIP reform on inpatient services for low-income populations." New key findings were added, including: primary hospitals facing moral hazard issues as policy dividends are released; secondary hospitals experiencing significant structural contradictions, showing a balancing act between cost control and quality; and tertiary hospitals addressing the rigid demand for continuity of care while improving quality and efficiency. Finally, policy recommendations were introduced, such as innovative tools like “outpatient-inpatient payment linkage” and “quality performance rebate mechanisms” to balance cost and quality.
2) **Introduction**: We expanded the discussion on global healthcare costs and equity issues, reinforcing the theoretical framework of the study and providing a more detailed review of previous research on healthcare payment reform, particularly in relation to the Chinese DIP model. Literature on the challenges and impacts of healthcare reform across different patient populations was also introduced. The innovative nature of the research question was clarified, and the academic contribution of the two-dimensional analytical framework of “hospital hierarchy-policy cycle” was highlighted.
3) **Discussion**: The discussion was systematically revised to incorporate the reviewers’ suggestions. We added “cross-regional case comparisons” (e.g., practices in Tai'an City and Guangdong Province) to deepen the analysis of the heterogeneity of reform effects. A tiered payment model is now proposed (bundled payment for primary hospitals and efficacy-based payment for tertiary hospitals), along with corresponding policy recommendations that directly address the reviewers’ concerns about “policy adaptability” and “moral hazard.” The behavioral motivations of healthcare providers and low-income patients were explored in a hierarchical and phased manner, and the limitations of the policies were identified, with suggestions for improvement.

**2. Principles for Responding to Review Comments**

We have addressed most of the reviewers' suggestions and clarified two elements that were not adjusted due to data or policy constraints.

1) **Missing outpatient data**: The “Limitations” section now clearly states that China’s DIP reform currently only covers inpatient services and that there is a lack of standardized regulation for outpatient data. However, we have committed to conducting a mixed-methods study in the future.
2) **Cross-regional generalizability**: By adding case comparisons with “Tai'an City” and other regions, the study indirectly demonstrates the transferability of the findings and commits to expanding the study across regions.

**3. Reasoning for Not Fully Adopting Some Recommendations**

Certain individual recommendations (e.g., “increase patient health outcome tracking” and “measure new payment models”) were not implemented due to the limitations of retrospective data and the scope of the study. However, we have responded as follows:

1) **Indirect evidence supplementation**: We have indirectly inferred changes in quality of care by analyzing the association between readmission rates and changes in admissions. We also suggest potential new payment models based on some of the evidence derived from this study.

We believe that the revised manuscript adequately addresses the reviewers' and editors' concerns. We remain fully committed to further revisions if additional suggestions are made.

**Response to reviewer 1:**

**The paper presents an interesting and relevant topic. However, it suffers from several drawbacks that need to be corrected before its possible acceptance.**

**1.The abstract needs to be rewritten, especially clarify the background, before objectives.**

- To address this comment, I revised the abstract to better clarify the background before introducing the objectives.

**Before revision:** The abstract starts with a detailed discussion of the DIP reform's impact, jumping directly into the results without clearly setting up the background.

**After revision:** I first introduce the global issue of rising healthcare costs and equity concerns, establishing the broader context for the DIP reform. I then proceed to introduce the objectives of the study in a clearer, more structured manner. I also ensured that the transition between the background and objectives is smoother.

Here is the specific revised portion:

1. The background now begins with a discussion on the global healthcare cost concerns and health equity issues, followed by an explanation of China's healthcare reform efforts, setting the stage for the introduction of the DIP reform.
2. The objectives are then stated clearly after this background, focusing on the goals of evaluating the DIP reform’s effects on inpatient services for low-income populations in S City.

This change can be found in the **Abstract section**, where I structured the first two sentences to reflect the broader context and the study’s specific aims more clearly.

**2. I advise the authors to professionally proofread their manuscript prior to resubmitting.**

- Thank you very much for the reviewer’s suggestion. I believe the feedback might refer to the data presentation section where I did not label the beta coefficients and reported p-values in an inconsistent manner. In response to the reviewer’s advice, I have made the following specific revisions to ensure proper formatting and clarity:

1. **Beta Coefficients Labeling:** I have added numbering to all beta coefficients to ensure they are clearly marked for easy reference throughout the results section.
2. **P-value Reporting:** As per the journal’s guidelines, I have revised the reporting of p-values. For p-values less than 0.001, I have now reported them as “p < 0.001”; for p-values greater than or equal to 0.001, I have reported the exact values. This ensures the p-values are reported in accordance with the journal's formatting standards.
3. **Abbreviation Formatting:** I have also reviewed the formatting of all abbreviations that first appear in the manuscript, ensuring that they are properly defined and formatted in accordance with the journal’s guidelines.

These revisions can be found in the **data results section** as well as in the **standardization of abbreviations**. You can refer to the version with track changes where these modifications are highlighted with **red added content** and **strikethroughs for deleted sections**.

**3.Introduction is very very short. In my opinion needs more literature to support the problem of the study**

- Thank you for the reviewer’s suggestion regarding the Introduction. I appreciate the feedback and have made the following revisions to address this concern:

I have expanded the **Introduction section** by incorporating more relevant literature to better support the research problem. Since there is currently no specific research on the DIP reform targeting low-income populations, I referenced studies that focus on other groups, such as women and children, as well as research on physician behavior, to enrich the content and provide a broader context for the study. Additionally, I included more references discussing the background of healthcare reforms globally and specifically in China, which helps contextualize the study and strengthen the foundation for understanding the significance of the DIP payment reform.

The expanded content includes:

1. A broader discussion of global healthcare cost concerns and equity issues.
2. A more detailed explanation of previous studies on healthcare payment reforms, particularly those related to the DIP model in China.
3. Studies on healthcare payment reforms focused on women and children.
4. Research examining how different payment models influence physician behavior, which can be analogous to the impact of the DIP reform on healthcare practices.
5. Literature on the challenges and impacts of healthcare reforms in different patient populations, providing insights that may be applicable to low-income groups.

These additions can be found in the **Introduction section** of the manuscript. Please refer to the version with track changes, where the newly added literature is highlighted in **red**.

**4.Methods chapter needs more specification of empirical work. Why this method?**

- I have added more specific details about the empirical work and provided a clearer justification for the choice of method used in this study. I expanded the explanation to include why the multi-stage ITS analysis was chosen as the most appropriate method for assessing the impact of the DIP payment reform.

The reasons for choosing this method are as follows:

1. **Multi-stage ITS analysis** is particularly well-suited for evaluating the effects of policies that are implemented incrementally, such as the DIP reform, which has distinct stages of intervention. This approach allows us to capture both the immediate and sustained impacts of the policy over time, providing a comprehensive understanding of its effects.
2. The **multi-stage ITS** method also allows for the analysis of variations in policy effects over time and across different hospital tiers, which is critical for understanding the heterogeneous impacts of the DIP reform. This is essential given the complexity of the reform and the varied responses of hospitals at different levels of care.
3. This method is particularly useful in quasi-experimental settings, where randomization is not possible, and provides a robust way to assess the impact of interventions like the DIP reform on healthcare outcomes for low-income populations.

These clarifications and justifications can now be found in the **Methods section** of the manuscript. Please refer to the version with track changes, where these additions are highlighted in **red**.

**5.The resultas are very complet, but need more discussion. Have results in another provinces?**

- I appreciate the feedback, and in response, I have made the following revisions to enhance the discussion and address the concern for more in-depth analysis:

1. **Expanded Discussion with Results from Other Provinces:**
   Since there is currently no specific study on low-income populations in other provinces, I introduced findings from studies conducted on the general population in different provinces. I compared these results with those from S City to explore whether similar trends are observed in different regions. This comparison adds more depth to the discussion and helps to contextualize the findings in a broader national framework.
2. **In-depth Analysis of Tiered Heterogeneity in the Impact of DIP Reform:**
   I further elaborated on the **tiered heterogeneity** in the impact of the DIP reform on inpatient service utilization for low-income patients. Specifically, I explored how the effects of the reform vary across different hospital tiers (primary, secondary, and tertiary hospitals) and discussed how each tier’s unique characteristics contribute to the observed differences. This part of the discussion highlights the complexity of the reform’s effects and provides a more nuanced understanding of its impacts.
3. **Mechanism Explanation:**
   To address the mechanisms behind the observed heterogeneity, I offered a preliminary explanation of how the DIP reform might lead to different responses across hospital tiers and patient groups. This explanation touches upon factors such as hospital resource availability, patient demand for care, and financial incentives, which help explain why the reform's effects are not uniform across all groups.

These changes significantly expand the **Discussion section**, providing a more detailed and thorough analysis of the results, including a comparison with studies from other provinces, a deeper exploration of tiered heterogeneity, and an initial discussion of potential mechanisms behind the observed effects. Please refer to the version with track changes, where the newly added content is highlighted in **red**.

**Other questions?**

**1- Why? January 1, 2019, to December 31, 2023.**

- The timeframe for this study was selected based on the following reasons:

1. **Data Availability and System Improvements:** After the establishment of China's National Healthcare Security Bureau in March 2018, provinces and cities progressively set up local healthcare security bureaus and enhanced their electronic healthcare information systems. In the study city, City S, Hubei Province, the local medical security bureau was officially established in November 2018. This was followed by the integration and standardization of the healthcare billing systems across the city’s hospitals. January 1, 2019, marked the official launch of the city's electronic healthcare billing database, which had previously been unavailable due to fragmented systems and the lack of unified management. As a result, the study period began in January 2019 to ensure data integrity and reliability.
2. **Methodological Requirements:** Interrupted time series analysis requires sufficient data points before and after the intervention to control for trend fluctuations and improve statistical validity. In this study, the DIP reform was divided into two phases: the Pilot phase (January 2021 to December 2021) and the Advancement phase (January 2022 to December 2023). December 31, 2023, was chosen as the cutoff date to include a complete 24 months of data for the Advancement phase. This ensured the ITS requirement of at least 8 time points after the intervention was met and allowed for the observation of long-term policy effects, thereby enhancing the scientific validity and robustness of the conclusions.
3. **Policy Implementation Process:** The DIP payment reform was launched in 2020 across 71 pilot cities in China. However, in S City, the reform did not fully cover all healthcare organizations until January 2022. By December 2023, the reform had been in place for two years, which is sufficient to capture the behavioral changes of hospitals, transitioning from "adaptive adjustments" to "strategic optimization," and to evaluate the sustainability of the policy’s effects.

**2- Why Hubei Province? and not other? Is special?**

- Hubei Province's S City was selected as the study population based on the following considerations:

1. Regional Representativeness: Hubei Province, located in central China, has an economic and social development level that is representative of the middle-income regions in the country. In 2023, S City's GDP reached 235.903 billion yuan (ranking seventh in the province), with a resident population of 3.15 million, making it a typical city in central China. Previous studies on the DIP reform have predominantly focused on cities in the eastern or western parts of China, such as Guangdong and Guizhou. There is a noticeable gap in studies analyzing the central region. This study fills that gap, offering new insights for a comprehensive assessment of the policy’s effects across China.
2. Suitability for Studying Low-Income Populations: S City has been a key area for poverty alleviation efforts, with 190,000 low-income individuals (annual income ≤ 20,000 yuan) residing in the city as of 2021. The city boasts 100% social insurance coverage and has jurisdiction over six national-level poverty-stricken counties, from which 810,000 people were successfully lifted out of poverty between 2016 and 2020. Given the city’s prominence in addressing healthcare needs and financial burdens for low-income populations, S City provides an ideal setting to observe the impact of healthcare payment reforms on vulnerable groups.
3. Policy Pilot Advantage: S City was selected as one of the first 71 pilot cities for the DIP reform in 2020. By January 2022, the city had achieved full coverage of the payment methods. Its healthcare insurance management practices have been recognized as a “National Advanced Unit of Healthcare Security,” ensuring high-quality data and standardized reform processes that truly reflect the effects of policy implementation. Additionally, S City’s three-tier healthcare network (comprising 6 tertiary hospitals, 44 secondary hospitals, and 214 primary hospitals) provides a comprehensive structure, offering a rich context for analyzing the heterogeneity of hospital tier responses to the reform.
4. Data Accessibility and Uniqueness: This study utilizes a hospitalization reimbursement database authorized by the S City Municipal Health Protection Bureau. The database covers 1.17 million hospitalization records from 2019 to 2023 for low-income individuals in the city. It contains detailed information on diagnoses, treatments, costs, and hospital tiers, providing an irreplaceable empirical foundation for a thorough investigation into the effects of payment reform on vulnerable populations.

In conclusion, S City’s representativeness within the central region, its low-income population characteristics, the city’s involvement in the policy pilot, and the high-quality data make it the ideal location for this study.

**Response to reviewer 2:**

**1.The focus on inpatient services alone narrows the lens through which the DIP reform’s impact is assessed. Many healthcare interactions, especially among low-income populations, occur in outpatient settings or general practices, where preventive care and chronic disease management play a critical role. The omission of these dimensions’ risks underestimates the full impact of the reform. Moreover, exclusion of outpatient data limits the study’s ability to evaluate whether reduced inpatient costs lead to cost-shifting or unintended consequences in other parts of the healthcare system. To what extent would the inclusion of outpatient and general practice data better capture the ripple effects of DIP reform, particularly in reducing reliance on costly inpatient care and promoting preventive health measures?**

Thank you for your insightful feedback on this critical issue. We fully agree that the role of outpatient services and primary care in chronic disease management and preventive care is crucial for a comprehensive assessment of the impact of DIP reform. In response to your suggestions, we offer the following clarifications:

1. **Limitations of Current Chinese Health Care Policy and Data Availability:** At present, China's DIP payment reforms primarily focus on inpatient services as the core pilot area, and outpatient services have not yet been incorporated into a standardized payment system(Tang et al., 2025; Zheng et al., 2024). More importantly, the current hospital information system only transmits inpatient data to the health insurance department in real time, while outpatient data are not monitored or managed in real time(Zhuanglang County People’s Government, 2021). This dual limitation—both in policy and technology—makes it challenging to establish causal links between the economic behaviors of outpatient services (e.g., potential cost-shifting or changes in preventive healthcare demand) using the available data(Jia et al., 2021).
2. **General Limitations of Existing Research:** This issue is not unique to our study. The authoritative literature in this field is similarly constrained by the lack of outpatient data, and as a result, most research focuses on inpatient services(Shi et al., 2024; Tang et al., 2023; Wu et al., 2023; Xie et al., 2022). We indirectly explored the impact of insufficient outpatient service capacity on the demand for inpatient hospitalization by analyzing heterogeneity at the stratified hospital level (e.g., the relationship between rising inpatient hospital costs and moral hazard in primary care hospitals; see Discussion, para. 5). This provides new evidence for understanding the systemic challenges of a tiered healthcare system.
3. **Commitment to Future Research:** We have explicitly acknowledged this limitation in the “Study Limitations” section and emphasized: "In the future, if outpatient services are included in policy and data become available, we will obtain outpatient data through ethical review and conduct a comprehensive analysis of the inpatient-outpatient linkage." Furthermore, we plan to design a mixed-methods approach (e.g., interviews with hospital administrators and surveys of patient behavior) for a follow-up study. This will allow us to fill the gaps in quantitative analysis with qualitative data and explore the potential mechanisms of cost-shifting and preventive care in greater depth.

Thank you again for encouraging us to consider the boundary conditions of our study design. Your suggestions point to an important direction for future research, and we will continue to monitor the evolution of China's health insurance policies and refine our analytical framework once the data become available.

**2.Focusing on a single pilot city in central China creates challenges in generalising the findings to other regions with different socioeconomic, cultural, and healthcare dynamics. Variability in healthcare infrastructure and policy implementation across regions could significantly alter the outcomes of similar reforms. Additionally, the urban-rural divide, prevalent in many parts of China, raises questions about the equity and scalability of these reforms in less resource-rich settings. How can research employ cross-regional comparative studies to identify context-specific facilitators and barriers to the successful implementation of DIP reforms?**

Thank you for your insightful comments regarding the external validity of our study. We fully acknowledge that differences in socioeconomic conditions, healthcare resources, and cultural practices across geographic regions of China may influence the effectiveness of DIP reform implementation. In response to your suggestions, we offer the following clarifications:

1. **Rationale for Focusing on a Single-City Case:** This study concentrates on City S because of its representativeness in the CPR pilot. As one of the first 71 DIP pilot cities in China, City S exhibits standardized characteristics in terms of policy implementation intensity and health insurance information system development(National Health Insurance Agency, 2021a). Additionally, its economic development (with a GDP of 23.59 billion yuan in 2023) is comparable to that of most prefectural-level cities in China(National Bureau of Statistics, 2023). The in-depth analysis of “typical cases” is a well-established approach in policy pilot studies, providing a direct reference for similar cities(He, 2023; Liao et al., 2024; Zhang et al., 2024). Moreover, the recognition of the S Municipal Health Insurance Bureau with the National Advanced Award in 2022 for its “outstanding results in hospitalization payment reform” further underscores the replicability of its experience(National Health Insurance Agency, 2021).
2. **Scalability of the Methodological Design:** While the study is based on data from a single region, we partially address the issue of regional variation through two approaches:
1) **Analysis of Heterogeneity at the Hospital Level:** Our findings indicate that tertiary hospitals offer more continuity of care for patients with high disease severity while effectively controlling costs. Secondary hospitals face pressure to manage costs and referrals, which leads them to adopt a strategy of “shorter hospitalization days + increased frequency.” In contrast, primary care hospitals are exposed to moral hazard and may struggle to control costs due to a lack of resources or capacity. This hierarchical difference could be analogous to regional disparities—hospitals in high-resource regions (e.g., eastern provinces) versus low-resource regions (e.g., rural western areas) may display similar behavioral divergences.
2) **Multi-Stage ITS Modeling:** By capturing the evolution of policy effects during the transition period (2021) and the deepening period (2022) (see Table 2), this study sheds light on the dynamic adaptation process of the policy. This provides valuable insights into the pace of policy implementation across different regions.

As mentioned in the “Limitations” section, we have committed to introducing mixed methods in subsequent studies. This will include interviews with hospital administrators (e.g., to explore hospitals' behavioral responses to policy reforms) and focus group discussions with patients (e.g., to assess low-income patients’ satisfaction with care and individual health outcomes), complementing the quantitative analyses to identify context-specific drivers.

Your suggestions have deeply informed our research planning. We believe that the proposed design will not only uncover the regional heterogeneity of DIP reforms but also provide a differentiated policy toolkit for national strategies, such as hierarchical diagnosis and treatment and precision health insurance payments.

**3.While the study demonstrates clear outcomes, such as changes in costs and readmission rates, it does not delve into the behavioural mechanisms driving these results. For instance, how are hospital administrators adapting their practices in response to financial incentives? Are low-income patients changing their healthcare-seeking behaviours due to reduced costs, or are they facing barriers that the study does not capture? Without understanding these dynamics, it is difficult to design interventions that address root causes. What qualitative methods, such as focus groups, interviews, or behavioural experiments, could be applied to unpack the mechanisms by which DIP reform influences decision-making among key stakeholders, including hospitals, physicians, and patients?**

Thank you for your insight into this key mechanistic issue. We fully agree that understanding the decision-making behaviors of hospital administrators, physicians, and patients is central to optimizing DIP reform. Although this study was limited by data availability and ethical approval, we responded to the exploration of behavioral mechanisms through the following design components:

Despite the lack of direct behavioral data, we provided important clues to decision-making mechanisms through multidimensional indicator correlation analysis and theoretical framework integration:

1. **Behavioral differences at the hospital level:**
1) **Tertiary hospitals:** a significant decrease in inpatient costs (β₅ = -0.035, P < 0.001), combined with their admitted case characteristics (Discussion paragraph 9), suggests that administrators responded to DIP payment constraints by optimizing clinical pathways (e.g., standardized care, resource pooling) and provided a more continuum of care for low-income, critically ill patients, which is consistent with DRG reforms' high resource hospitals' strategies(Ding et al., 2023; Zou et al., 2020).
2) **Secondary hospitals:** the “shorter days + more frequent” strategy (0.14 days/month decrease in LOS, β₃ = 0.122, P = 0.020) resulted in higher readmission rates, suggesting that administrators may sacrifice treatment integrity under cost containment pressures (Discussion, paragraph 8). This finding contrasts with the phenomenon of “fragmented hospitalization” in the European DRG study(Böcking et al., 2005; Busse et al., 2013; Fässler et al., 2015), and highlights the unique adaptations of hospitals under China's tiered care system.
3) **Primary hospitals:** when the out-of-pocket ratio falls below the 5% threshold, the demand for non-acute hospitalization surges (57.14% of hospitalizations in primary hospitals), directly confirming Arrow's theory of moral hazard(Arrow, 2001), but manifesting itself as “primary concentration” rather than generalized overmedicine (Discussion, paragraph 5).
2. **Patient behavioral inferences:** Low out-of-pocket ratios (OOP ≤5%) with inadequate outpatient reimbursement further impacts patient readmissions at primary care hospitals, suggesting that low-income patients are more price sensitive(Bao and Wang, 2023; Liao et al., 2024). This finding provides a quantitative basis for designing differential reimbursement rates.
3. **Theoretical mapping across regional cases：**We have added comparisons with the DIP pilot regions of Tai'an City and Guangdong Province to the discussion(Lai et al., 2022; Shi et al., 2024).
1) **Tai'an city:** CMI increase and hospitalization ratio decrease in tertiary hospitals, confirming the hierarchical diagnosis and treatment effect of “upward movement of serious illnesses and downward movement of less serious illnesses” in this study (Discussion, paragraphs 9 and 10).
2) **Guangdong Province:** Secondary hospitals control costs through “referral coordination” rather than simply shortening hospitalization days, suggesting that regional resource distribution may modulate hospital strategies (Discussion, paragraph 8).
4. **Future Mixed Methods Study Design.**We have promised in the Limitations section:
1) In-depth interviews with hospital administrators: focusing on clinical pathway optimization in tertiary hospitals, performance evaluation pressures in secondary hospitals, and resource allocation decisions in primary hospitals.
2) Patient focus groups: to explore the thresholds at which changes in out-of-pocket expenses affect healthcare choices, stratified by income (e.g., low-income households vs. marginal low-income groups).

Your suggestion opens the “black box” of mechanism research. As a next step, we will apply for ethical approval for cross-regional health insurance data sharing, and combine the quantitative framework of this paper with the qualitative tools mentioned above to construct a full chain model of “payment incentives-behavioral responses-system outcomes”.

**4.The study focuses predominantly on hospitalisation costs and 30-day readmission rates, so it offers a limited view of the reform's impact. Broader patient-centred outcomes, such as overall health improvement, quality of care, and patient satisfaction, remain to be explored. These metrics are key to assessing whether cost reductions are made at the expense of the quality of care or whether patients gain tangible health benefits. To what extent do the results guarantee and respond to the impact of the reform?**

Thank you for your deep concern about patient-centered outcomes. We fully recognize that health improvement, quality of care, and patient satisfaction are the core dimensions for assessing the social benefits of payment reform. In response to your suggestions, we offer the following clarification:

Because this study is based on retrospective data, we were unable to include patient-centered outcomes such as patient satisfaction and current health status. Due to data collection limitations, we were only able to analyze using existing metrics such as hospitalization costs and readmission rates. Despite the limitations of retrospective data, we provided important clues to quality of care and health outcomes through multilevel metric correlation analyses:

1. **Readmission Rates and Completeness of Care:** A significant increase in secondary hospital readmission rates (β₃ = 0.472, *P* < 0.001) occurred in tandem with shorter hospitalization days (LOS decreased by 0.14 days/month), suggesting an “efficiency-quality trade-off” (Discussion, para. 8). This finding contrasts with the DRG study's conclusion that “fragmented hospitalization leads to care breakdown”(Choi et al., 2019; Li et al., 2022; Liu et al., 2024), suggesting a unique adaptation strategy for Chinese secondary hospitals under the pressure of tiered care. The increase in readmission rates in tertiary hospitals (β₃ = 0.685, *P* < 0.001) stems mainly from the rigid demand for treatment of critically ill patients (e.g., chemotherapy for oncology) (Discussion, paragraph 9), reflecting the release of rigid demand for health care by policy rather than a decline in quality.
2. **Out-of-pocket thresholds and health accessibility:** The surge in demand for nonacute hospitalizations (57.14% of hospitalizations) when the out-of-pocket percentage for primary hospitals falls below 5% is direct evidence of the high price sensitivity of low-income patients (Weissman et al., 1991). Although it may raise moral hazard, it also suggests that the policy significantly lowered economic barriers and allowed more patients (especially the chronically ill) to access inpatient services (Discussion, paragraph 6), indirectly suggesting improved health accessibility. However, it cannot be ruled out that tertiary hospitals may have provided poor quality services to some extent due to lack of resources or capacity, which may also have led to readmission of patients(Shen et al., 2020; Wang and Zhang, 2023; Yip et al., 2019).
3. **Evidence on the balance between cost containment and quality assurance：**The study found that tertiary hospitals achieved a reduction in hospitalization costs (β₅ = -0.035) in the deepening period (after 2022) while accepting patients with rising disease difficulty (citing comparative data from Tai'an City, Discussion paragraph 9), suggesting that they achieved their cost control goals through clinical pathway optimization (e.g., standardized care, resource pooling) rather than at the expense of quality to reach cost-control goals. This is consistent with the practice of “value-based care” in international research (Navathe and Liao, 2022).

In the short term, we will submit a policy proposal to the S Municipal Health Insurance Bureau to encourage hospitals to embed a standardized patient satisfaction questionnaire (covering dimensions such as diagnosis and treatment outcomes, communication quality, and cost transparency) in the discharge process.

We will also apply for ethical approval to conduct joint patient-physician focus groups to explore the impact of changes in patient out-of-pocket payments on delay in access to care, treatment adherence, and how DIP payment standards affect physicians' clinical decision-making (e.g., selection of screening tests, grasping of indications for hospitalization).

**5.The reduction in hospitalisation costs, particularly in secondary and tertiary hospitals, indicates that PID reforms can ease the financial burden on low-income populations. However, the cost increases observed in primary hospitals suggest potential inefficiencies in the allocation of resources, possibly due to overutilization or increased administrative pressures on lower-level establishments. How does the study respond to disparities in the allocation of resources between hospital levels in order to ensure that cost reductions in higher-level establishments do not inadvertently shift the burden onto primary hospitals?**

- We would like to sincerely thank the reviewers for their thoughtful and insightful review of our research. Your question regarding the increase in primary hospital costs has drawn attention to potential challenges in the implementation of the DIP reforms and prompted us to reflect more deeply on the efficiency of resource allocation. We apologize for not providing a more detailed response to this concern earlier, as we did not elaborate sufficiently on the design and implementation of the DIP reforms in this article. Below is our detailed response to address your question:

1. **Design and Implementation Background of DIP Reform**: Under the DIP payment reform, the total Medicare fund allocated to hospitals is capped each year, leading to intense competition among hospitals, particularly between Level II and Level III hospitals. These hospitals often target patients from Level I hospitals in an attempt to maximize revenue. Specifically, the payment coefficients for the same disease vary by hospital tier, typically increasing with hospital level. This means that tertiary and secondary hospitals can treat common diseases at a lower cost while benefiting from higher reimbursement rates. As a result, these hospitals often prioritize treating common diseases, leaving Level I hospitals struggling to survive, especially if they lose patients to higher-level institutions.
2. **Survival Pressure and Resource Allocation in Primary Hospitals**: Given the above context, primary hospitals are under greater economic strain, not necessarily due to cost-cutting or burden transfer from secondary and tertiary hospitals, but due to the payment disparities between different hospital levels. After the implementation of the DIP reform, grassroots hospitals may experience reduced income because of the differences in payment coefficients. To address the concern of secondary and tertiary hospitals "siphoning" patients from grassroots hospitals, the S Municipal Medical Insurance Bureau took proactive measures. After extensive calculation and demonstration, 363 “grassroots diseases” were identified—these include common, frequently occurring, and chronic diseases suitable for treatment at primary medical institutions, aligning with the functional capacities of grassroots hospitals. As part of the DIP reform, a unified payment standard for these diseases was introduced across all hospital levels, aiming to promote tiered diagnosis and treatment. This policy ensures that patients can select the most appropriate medical institution for their needs while preventing over-reliance on higher-level hospitals.
3. **Efficiency of Resource Allocation in Grassroots Hospitals**: Despite these efforts, the rising costs in primary hospitals still highlight inefficiencies in resource allocation. This indicates that, although the Medical Insurance Bureau has designed a policy framework for grassroots hospitals, challenges in resource allocation remain. Specifically, grassroots hospitals may struggle to control costs effectively due to insufficient resource input or management capacity. These issues underscore the need for further improvements in future policy design and resource distribution. We believe these challenges stem not only from the payment reform mechanisms but also from factors such as internal hospital management, equipment allocation, and staffing.
4. **Policy Suggestions and Future Research Directions**: To address this issue, we suggest that policymakers increase resource investment and support for grassroots hospitals, particularly in areas such as management capacity, equipment allocation, and personnel training. Furthermore, the Medical Insurance Bureau could strengthen the guidance on tiered diagnosis and treatment to ensure that grassroots hospitals can reduce costs and improve efficiency through more rational resource allocation and capacity-building. In our policy recommendations, we propose that the S Municipal Medical Insurance Bureau enhance support for grassroots hospitals in future reforms and ensure fairness in resource allocation through robust monitoring and evaluation mechanisms.

Once again, we appreciate your profound questions and suggestions regarding our research. Your input has been invaluable in prompting us to think more critically about the reform's implementation details and areas for improvement. In future studies, we will continue to focus on this issue and explore ways to optimize the resource allocation mechanisms for hospitals at all levels, ensuring that the DIP reform better serves hospitals, particularly those at the grassroots level.

**6.Although IDP reforms have improved access for low-income patients, rising readmission rates suggest inefficiencies, such as inadequate post-discharge care or premature discharges motivated by cost-containment pressures. This emphasises the tension between improving accessibility and maintaining system efficiency. What specific strategies can be implemented to reduce readmissions while preserving the accessibility gains achieved by the PID reforms?**

**7.The potential for moral hazard arises when cost-cutting encourages overutilization of inpatient services. For example, patients may seek hospital care for illnesses that could be treated on an outpatient basis, overloading hospital resources. Similarly, hospitals may prioritise volume over quality in order to maintain their revenues under revised payment structures. How does the study contribute to understanding and defining reimbursement models that mitigate moral hazard, such as value-based payment systems or incentives for care coordination, to promote efficient use of resources?**

Thank you for your insight into the issue of balancing efficiency and equity in DIP reform. We note that comments 6 (readmission rate and accessibility paradox) and 7 (moral hazard and payment model optimization) are closely related in nature - both focus on how to achieve quality of care and efficient use of resources under the goal of cost containment through payment tool innovation. Therefore, we combine the two comments to systematically describe the integrated strategic framework of this study for the triple objective of “efficiency-quality-equity”.

This study reveals that the driving mechanism for the increase in readmission rates varies by hospital tier or functional position (Table 2, Figure 3):

1. **For primary hospitals:** increased reimbursement rates → weakened patient cost perception → demand release (including reasonable demand and moral hazard). At the same time, studies have shown that the DIP payment reform further promotes hierarchical diagnosis and treatment, but policy makers have not given primary hospitals sufficient resources, manpower allocation, etc., in guiding this trend, resulting in high costs and lack of cost control in primary hospitals after the reform. This will lead to the primary hospitals to avoid the risk of loss and the health insurance department's punishment and make corresponding unfavorable coping strategies for patients. For the above problems, this study proposes the following strategies:
1) Outpatient-hospitalization payment linkage: For chronic diseases such as hypertension and diabetes, design a bundled payment package of “annual outpatient management + limited number of hospitalizations” to guide primary hospitals to prioritize preventive interventions.
2) Enhanced reimbursement rates and coverage for outpatients will ensure that patients receive adequate financial support for outpatient treatment, thereby reducing unnecessary hospitalizations.
3) Improving the service capacity of primary care by investing in medical equipment, drug supply and human resources is essential to ensure quality chronic disease management.
4) Setting clear hospitalization thresholds and audit mechanisms can ensure that hospitalization is medically necessary and help prevent over-hospitalization due to financial incentives.
2. **For secondary hospitals:** payment standard constraints → hospital revenue maximization strategies → alienation of service behaviors (including quality trade-offs and multiple admissions). This leads directly to a significant increase in readmission rates, suggesting that efficiency-oriented cost-control measures may lead to incomplete treatment. However, what also needs to be taken into account is that in China, due to the special status of secondary hospitals, they not only face pressure for payment reform, but also have referral responsibilities (Hu et al., 2023; Wang et al., 2017; Wu et al., 2023). This may mean that their behavior goes beyond simply breaking up hospitalizations, but that they need to find a balance between controlling costs and maintaining the quality of care. For example, shorter hospitalization days may help control costs, but without effective outpatient or community care support, patients may need to be rehospitalized, leading to higher readmission rates. At the same time, as referral hubs, secondary hospitals may need to refer some complex cases to tertiary hospitals while receiving cases from primary hospitals, adding to the complexity of management. **Responding to strategy recommendations:**
1) Pure cost-control metrics (e.g., days of hospitalization) may induce unintended consequences, and quality hedges need to be put in place (e.g., incorporating readmission rates into payment adjustment factors)
2) The referral hub function of secondary hospitals requires dedicated reimbursement to avoid financial imbalances due to the fulfillment of public duties.
3) Additional payments are made on a case-by-case basis for cases where follow-up is completed within 72 hours of discharge.
3. **For tertiary hospitals:** the rise in readmission rates stems mainly from the rigid demand for treatment of critically ill patients (e.g., chemotherapy for oncology) (Discussion, paragraph 9). This trade-off relationship suggests that judging service quality solely on readmission rates may underestimate the health benefits of the policy, and that a multidimensional evaluation system incorporating treatment integrity is needed in the future. Strategy Recommendation:
1) Exempt specific conditions from readmission penalties: Create a whitelist of 'planned readmissions' for conditions such as oncology that require cyclical hospitalization to avoid policy missteps. More specifically, create a 'Critical Care Continuity Payment Module' to consolidate the billing of planned readmissions within a six-month period to avoid statistical distortions caused by disaggregated hospitalizations.

**8.The dual impact of PID reforms emphasises the need for policies that strike a balance between cost containment and equitable access. Strengthened regulatory oversight, coupled with investments in outpatient and community-based care, could address the inefficiencies and unintended consequences highlighted by the study. In addition, the adaptability of health insurance reforms to different contexts requires further research to ensure their wider applicability. What innovative frameworks, including bundled payments or capitation models, could be tested to optimise the balance between cost control, quality improvement, and equitable access to healthcare?**

Thank you for your insights into the multidimensional balancing issues in DIP reform. The innovative framework you propose is an important direction for optimizing DIP reform. Although the core objective of this study is to assess the direct impact of DIP reforms on inpatient services for low-income populations, and this study is limited by the scope of the policy pilots and data boundaries, we offer the following transferable design principles for these frameworks through the analysis of heterogeneity in inpatient services:

1. Threshold sensitivity management: Identification of out-of-pocket thresholds (OOP ≤5%) for primary hospitals provides a basis for calibrating the “risk adjustment mechanism” in capitation payments.
2. Dynamic adjustment of payments: Differentiation of readmission rates for quality of care issues or continuity of care needs, with payments for secondary hospitals incorporating key factors such as readmission rates and patient health outcomes, and readmission rate assessments for tertiary hospitals incorporating consideration of continuity of care needs.

We are committed to systematically testing the adaptability of these frameworks in subsequent studies by obtaining data on innovative payment reforms in more regions through ethical approval. Your suggestions provide critical direction for this program and are appreciated.

**References**

Arrow, K.J., 2001. Uncertainty and the welfare economics of medical care. 1963. Journal of health politics, policy and law 26, 851–83. https://doi.org/10.1215/03616878-26-5-851

Bao Z., Wang Z., 2023. How Does the Tilted Triple Medical Security Policy Affect the Demand for Medical Care among Low-income Population? —Concurrently on Preventing from Large-scale Risk of Relapse to Poverty due to Illness. Insurance Studies 428, 97–111. https://doi.org/10.13497/j.cnki.is.2023.12.008

Böcking, W., Ahrens, U., Kirch, W., Milakovic, M., 2005. First results of the introduction of DRGs in germany and overview of experience from other DRG countries. J. Public Health 13, 128–137. https://doi.org/10.1007/s10389-005-0103-4

Busse, R., Geissler, A., Aaviksoo, A., Cots, F., Hakkinen, U., Kobel, C., Mateus, C., Or, Z., O’Reilly, J., Serden, L., Street, A., Tan, S.S., Quentin, W., 2013. Diagnosis related groups in europe: Moving towards transparency, efficiency, and quality in hospitals? BMJ 346, f3197–f3197. https://doi.org/10.1136/bmj.f3197

Choi, J.W., Kim, S.-J., Park, H.-K., Jang, S.-I., Kim, T.H., Park, E.-C., 2019. Effects of a mandatory DRG payment system in south korea: Analysis of multi-year nationwide hospital claims data. BMC Health Serv. Res. 19, 776. https://doi.org/10.1186/s12913-019-4650-8

Ding, Y., Yin, J., Zheng, C., Dixon, S., Sun, Q., 2023. The impacts of diagnosis-intervention packet payment on the providers’ behavior of inpatient care—evidence from a national pilot city in China. Front. Public Health 11, 1069131. https://doi.org/10.3389/fpubh.2023.1069131

Fässler, M., Wild, V., Clarinval, C., Tschopp, A., Faehnrich, J., Biller-Andorno, N., 2015. Impact of the DRG-based reimbursement system on patient care and professional practise: Perspectives of swiss hospital physicians. SWISS MEDICAL WEEKLY 145. https://doi.org/10.4414/smw.2015.14080

He, A.J., 2023. Scaling-up through piloting: Dual-track provider payment reforms in China’s health system. Health Policy Plan. 38, 218–227. https://doi.org/10.1093/heapol/czac080

Hu, H., Wang, R., Li, H., Han, S., Shen, P., Lin, H., Guan, X., Shi, L., 2023. Effectiveness of hierarchical medical system policy: An interrupted time series analysis of a pilot scheme in China. Health Policy Plann. 38, 609. https://doi.org/10.1093/heapol/czad018

Jia, L., Meng, Q., Scott, A., Yuan, B., Zhang, L., 2021. Payment methods for healthcare providers working in outpatient healthcare settings. Cochrane Database Syst. Rev. 2021, CD011865. https://doi.org/10.1002/14651858.CD011865.pub2

Lai, Y., Fu, H., Li, L., Yip, W., 2022. Hospital response to a case-based payment scheme under regional global budget: the case of guangzhou in china. Soc. Sci. Med. 292, 114601. https://doi.org/10.1016/j.socscimed.2021.114601

Li, Xing, Zhang, Y., Zhang, X., Li, Xinyan, Lin, X., Han, Y., 2022. Effects of fee-for-service, diagnosis-related-group, and mixed payment systems on physicians’ medical service behavior: Experimental evidence. BMC Health Serv. Res. 22, 870. https://doi.org/10.1186/s12913-022-08218-5

Liao C., Jin O., Hui W., 2024. Medical Service Value Enhanced by Medicare DRG Payment Reform: Evidence From H City. Research on Financial and Economic Issues 488, 89–103. https://doi.org/10.19654/j.cnki.cjwtyj.2024.11.007

Liu, Y., Wang, G., Qin, T.-G., Kobayashi, S., Karako, T., Song, P., 2024. Comparison of diagnosis-related groups (DRG)-based hospital payment system design and implementation strategies in different countries: The case of ischemic stroke. Biosci. Trends 18, 1–10. https://doi.org/10.5582/bst.2023.01027

National Bureau of Statistics, 2023. Annual GDP data for major cities [WWW Document]. URL https://data.stats.gov.cn/easyquery.htm?cn=E0105 (accessed 2.6.25).

National Health Insurance Agency, 2021a. Circular of the National Health Security Bureau on the Issuance of the Three-Year Action Plan for the Reform of DRG/DIP Payment Methods [WWW Document]. URL https://www.nhsa.gov.cn/art/2021/11/26/art_104_7413.html (accessed 2.19.25).

National Health Insurance Agency, 2021b. Office of the National Health Security Administration on the issuance of the list of DRG/DIP payment demonstration sites [WWW Document]. URL https://www.nhsa.gov.cn/art/2021/12/17/art_104_7504.html (accessed 1.29.25).

Navathe, A.S., Liao, J.M., 2022. Aligning value-based payments with health equity: A framework for reforming payment reforms. JAMA 328, 925–926. https://doi.org/10.1001/jama.2022.14606

Shen, M., He, W., Li, L., 2020. Incentives to use primary care and their impact on healthcare utilization: Evidence using a public health insurance dataset in China. Soc. Sci. Med. 255, 112981. https://doi.org/10.1016/j.socscimed.2020.112981

Shi, H., Cheng, Z., Liu, Z., Zhang, Y., Zhang, P., 2024. Does a new case-based payment system promote the construction of the ordered health delivery system? Evidence from a pilot city in China. Int. J. Equity Health 23, 55. https://doi.org/10.1186/s12939-024-02146-y

Tang L., Liang Q., Liu M., Xie F., Guo Z., 2025. The impact and evaluation of DRG policy on the hierarchical medical system in public hospitals. Chinese Hospitals 29, 50–55. https://doi.org/10.19660/j.issn.1671-0592.2025.2.11

Tang, X., Zhang, X., Chen, Y., Yan, J., Qian, M., Ying, X., 2023. Variations in the impact of the new case-based payment reform on medical costs, length of stay, and quality across different hospitals in China: An interrupted time series analysis. BMC Health Serv. Res. 23, 568. https://doi.org/10.1186/s12913-023-09553-x

Wang, Y., Sun, L., Hou, J., 2017. Hierarchical medical system based on big data and mobile internet: A new strategic choice in health care. JMIR Medical Informatics 5, e6799. https://doi.org/10.2196/medinform.6799

Wang, Z., Zhang, G., 2023. Primary hospitals in China: Current state, challenges, and solutions. Hepatobiliary Surg. Nutr. 12, 909–911. https://doi.org/10.21037/hbsn-23-571

Weissman, J.S., Stern, R., Fielding, S.L., Epstein, A.M., 1991. Delayed access to health care: Risk factors, reasons, and consequences. Ann. Intern. Med. 114, 325–331. https://doi.org/10.7326/0003-4819-114-4-325

Wu, Q., Zhao, Z., Xie, X., 2023. Establishment and application of the performance appraisal system for hierarchical diagnosis and treatment in China: A case study in fujian province. FRONTIERS IN PUBLIC HEALTH 11. https://doi.org/10.3389/fpubh.2023.1008863

Xie, H., Cui, X., Ying, X., Hu, X., Xuan, J., Xu, S., 2022. Development of a novel hospital payment system – big data diagnosis & intervention packet. Health Policy OPEN 3, 100066. https://doi.org/10.1016/j.hpopen.2022.100066

Yip, W., Fu, H., Chen, A.T., Zhai, T., Jian, W., Xu, R., Pan, J., Hu, M., Zhou, Z., Chen, Q., Mao, W., Sun, Q., Chen, W., 2019. 10 years of health-care reform in China: Progress and gaps in universal health coverage. Lancet 394, 1192–1204. https://doi.org/10.1016/S0140-6736(19)32136-1

Zhang, Y., Xu, S., Tan, G., 2024. Unraveling the effects of DIP payment reform on inpatient healthcare: Insights into impacts and challenges. BMC Health Serv. Res. 24, 887. https://doi.org/10.1186/s12913-024-11363-8

Zheng X., Zhou Y., Shen K., Xiao X., Chen S., Kang Z., Meng X., 2024. Analysis of the characteristics and hospitalization cost structure of high-ratio DRG cases. Chinese Hospitals 28, 10–13. https://doi.org/10.19660/j.issn.1671-0592.2024.4.03

Zhuanglang County People’s Government, 2021. How should medical insurance supervise the decomposition of inpatient expenses to outpatient service? [WWW Document]. URL http://www.gszhuanglang.gov.cn/zfxxgk/bmxzxxgk/bmxxgk/zlxylbzj/fdzdgknr/QTXX/art/2022/art_6943f121f82b47c3b2718a96a3a39f62.html (accessed 2.22.25).

Zou, K., Li, H.-Y., Zhou, D., Liao, Z.-J., 2020. The effects of diagnosis-related groups payment on hospital healthcare in China: A systematic review. BMC Health Services Research 20, 112. https://doi.org/10.1186/s12913-020-4957-5
